# Paying attention to attention: a program evaluation of faculty-delivered mindfulness-based attention training to optimize wellness and professionalism in medical students

**DOI:** 10.1186/s12909-024-05119-5

**Published:** 2024-02-23

**Authors:** Chloe Zimmerman Gunsilius, Malena M. Price, Scott L. Rogers, Ellen Flynn, Amishi P. Jha

**Affiliations:** 1 Department of Neuroscience, Brown University, 185 Meeting Street, Providence, RI 02912 USA; 2 Robert J. and Nancy D. Carney Institute for Brain Science, Brown University, Providence, RI USA; 3 Warren Alpert School of Medicine, Brown University, Providence, RI USA; 4 Department of Psychology, University of Miami, Florida, USA; 5 Mindfulness in Law Program, School of Law, University of Miami, Florida, USA; 6 Department of Medicine, Warren Alpert School of Medicine, Brown University, Providence, RI USA; 7 Department of Psychiatry & Human Behavior, Warren Alpert School of Medicine, Brown University, Providence, RI USA

**Keywords:** Mindfulness, Burnout, Resilience, Medical education, Executive function, Attention, Physician distress, Well-being, Professionalism, Mindfulness based attention training

## Abstract

**Background:**

As physician distress rises, medical schools must provide programs to counter such distress at the earliest stages of training. Mindfulness training (MT) is one intervention that can alleviate stress during medical school. However, framing MT around wellness alone misses the opportunity to connect core cognitive and psychological capacities strengthened by MT to professional goals and skill acquisition inherent to successful medical training. Here, we highlight how the attentional components of MT align with students’ goals of becoming *attending* physicians while promoting academic, psychological, and interpersonal flourishing. MT courses that focus on strengthening attentional capacities can intuitively link academic and professional development with wellness, appealing to a wide array of students.

**Methods:**

We iteratively recontextualized an existing short-form mindfulness training program for high-stress pre-professionals, known as Mindfulness Based Attention Training (MBAT), to the medical school context (MBAT-Rx). MBAT-Rx was offered by physician trainers to first-year medical students at Warren Alpert Medical School of Brown University as a tool for improving study habits and focus in addition to the development of both self-care and patient care strategies. MBAT-Rx consists of weekly, two-hour sessions over four weeks, with 10–15 min of daily mindfulness practice between sessions. At the end of the four weeks, students submitted voluntary program evaluation responses detailing their experience of the program.

**Results:**

Optional program evaluation responses (*n* = 67) highlight that students found the program to be useful for their academic success and ability to pay attention, their interpersonal relationships, and their psychological health. By framing MT as an opportunity to boost core attentional capacities and connecting this to professional and academic goals in addition to wellness, MBAT-Rx appealed to a wide variety of students.

**Conclusions:**

Our ongoing work suggests that framing MT as both a professional development and wellness promotion tool, taught by physicians themselves, and structured around students’ time demands, may be a successful model for medical schools looking to increase the impact of their mindfulness offerings. Such programs are needed to equip medical students to navigate the demands of a challenging healthcare training landscape.

## Background

Medicine is facing a crisis of distress within its workforce. Medical professionals are at elevated risk for burnout compared to other US working adults [1]. This risk has been further heightened recently by the extraordinary demands placed on physicians during the COVID-19 pandemic [2]. Physician distress often has professional consequences, as medical errors are more likely to be committed by physicians who self-report a higher burden of burnout symptoms [3–5]. In addition, patient satisfaction and trust are eroded by negative interpersonal interactions with disgruntled medical professionals [6]. Together, these performance and interpersonal challenges form a noxious feedback loop and further heighten physician distress, as evidenced by high depression and anxiety levels and increased risk of suicide among physicians, residents, and medical students [5, 7, 8].

The precursors of physician distress can be traced back to the earliest stages of medical education and training. Although trainees begin medical school with psychological health profiles on par with age-matched members of the general population [9], their profiles significantly diverge over subsequent years, driven primarily by increases in rates of depression and suicidal ideation [8, 10]. Such findings lead to an obvious question: How can future physicians be best trained to navigate the competing psychological, interpersonal, and professional stressors inherent within the medical field, while maintaining optimal wellness and professional conduct?

In recent years, mindfulness training (MT) programs aiming to improve student wellness have become more readily available at medical schools [11–15]. Mindfulness is often defined as “moment-to-moment, non-judgmental awareness, cultivated by *paying attention* in a specific way… as non-reactively, as nonjudgmentally, and as open-heartedly as possible” [16]. One manualized, frequently-offered, MT program, Mindfulness-Based Stress Reduction (MBSR), has been shown to benefit medical students’, residents’, and physicians’ mental and emotional health [13]. Findings demonstrate that MBSR significantly dampens depression, anxiety, fatigue, stress, and burnout symptoms, while increasing prosocial emotions such as empathy and compassion [12, 17–21].

Central features of mindfulness practice emphasize the ability to pay attention, nonjudgmentally, in the present moment [16]. Such abilities are essential to the *developing physician*’s professional goals. For example, one must learn how to pay attention nonjudgmentally to whatever a patient discloses, while also attending to the demands of comprehensively documenting this disclosure, accessing previous knowledge to formulate a diagnosis and treatment plan, and working within the dynamics of an integrated healthcare team. Developing the capacity to hold these competing sources of information in mind, without unnecessary rumination or distraction, is an important cognitive skill all future physicians must develop. When properly contextualized to the goals of medical training, MT may thus directly influence professional development. Prior findings within the psychology literature suggest that motivation to engage in behaviors is higher when these behaviors satisfy multiple goals simultaneously rather than a single goal [22]. In line with these findings, MT courses framed to include superordinate academic and professional goals, versus psychological and interpersonal well-being alone, may have greater appeal for medical students.

The medical training environment places significant cognitive demands on medical trainees, as they master didactic material, technical skill acquisition, and patient care. These cognitive demands impact trainees on multiple levels: psychological, interpersonal, and professional. Importantly, medical students report that academic performance is highly valued and considered relevant to their psychological well-being over time. They also consistently report that intensive academic demands are a significant barrier to their wellness [23]. Attention and associated executive functions are critical for successful academic performance as well as professional success as “Attending” Physicians [24, 25]. Attentional lapses are known to correspond with psychological health challenges, [26] and increase medical errors in medical trainees [27]. Without intervention, attentional lapses have been previously linked to performance errors [28]. Within the medical context, such lapses may contribute to instances of patient harm.

Mindfulness practice explicitly engages attention. Attentional processes and the psychological, interpersonal, and professional domains they affect are highly vulnerable to degradation amidst the ongoing stressors [29] common to medical education [30]. Empirical studies investigating the effects of MBSR, or a related program, Mindfulness-Based Cognitive Therapy (MBCT) among medical students, indicate that MT may improve attentional outcomes [31], problem-focused coping [32], and the ability to manage the challenging workload demanded by medical education [33]. A growing body of research indicates that MT is also highly effective for improving attentional performance across other high-demand professional training settings including among soldiers during pre-deployment intervals [34], university football players during pre-season training, [35] and undergraduates during the academic semester [36]. For this reason, attentional processes represent an important target for intervention in any prolonged high-stress cohort. Practices that preserve attentional functioning may have a multifaceted impact on academic success, professional development, and psychological health alike.

Despite MT’s demonstrated ability to benefit functions critical for success in medical training, many practical barriers pose challenges to effective implementation within medical schools. Two primary challenges, *time demands and contextualization,* may hamper medical students’ engagement with MT.

First, *issues of time demands:* MT offered to medical trainees should fit within the small pockets of time medical students have outside of their daily responsibilities. The popular MBSR program requires participation in an 8-week class with 2 to 2.5-h weekly class meetings, as well as 45-min daily “homework” practices that must be completed out-of-class [37]. This program requires a time commitment that is often infeasible for medical students’ schedules, limiting participation. Fewer and shorter weekly meetings with reduced minutes of daily practice may achieve greater participation among trainees. However, too few training hours spaced too far apart in time may not offer any real benefit to participants, as evidenced by a recent study that demonstrated the null effects of a novel, short-form mindfulness program for pediatric residents on burnout, empathy, or overall mindfulness [38]. For maximum impact, short-form MT programs must tightly balance scheduling demands with sufficient mindfulness practice exposure at the ideal time in medical education.

Second, *issues of contextualization*: MT should be contextualized for professional and educational goals, in addition to psychological and wellness improvements [12, 39]. While MBSR has been shown to benefit wellness outcomes in medical trainees, this program is frequently not offered by trainers who are embedded within the medical context, and thus, they may not be privy to the specific challenges medical students face. Short-form mindfulness programs specifically contextualized to the medical training setting and taught by physicians with medical education context familiarity may have greater impact on students’ practice engagement.

Here, we describe our implementation of a 4-week Mindfulness-Based Attention Training (MBAT) course for first-year medical students at the Warren Alpert School of Medicine of Brown University. MBAT is a short-form MT program developed to address the challenges of time pressure and context specificity inherent in high-stress professions [34, 40]. MBAT explicitly focuses on the use of mindfulness practices to develop core capacities of attention. Core themes highlight how attention is affected by stress, and its role in psychological health, academic success, and professional development. The program requires a two-hour session with a trainer for each of the four weeks of the course. Participants are encouraged to complete 15 min of out-of-class MT practices daily, for a minimum of 3–5 days per week. This program additionally employs a train-the-trainer (TTT) dissemination model in which context-familiar trainers are rapidly trained to deliver the program to their respective workplaces. To date, the core MBAT program has been adapted for a variety of occupational settings [41, 42].

## Methods

### Development of MBAT to preserve attentional and psychological capacities in high-stress professional training environments

A distinguishing and central feature of MBAT is its emphasis on the use of domain-contextualized mindfulness instruction and practices to promote attentional functioning necessary for specific professional training environments, while also supporting optimal psychological health. MBAT explicitly aligns learning and professional goals with wellness training. This alignment may increase receptivity and interest from a broader range of participants in medical education settings. That is, some medical students may resist participating in an MT program because of skepticism regarding the wellness components in the traditional framing of mindfulness. Yet, these same students may be receptive to participating if the program is framed as a way to improve learning a skill that could bolster their professional performance [23]. By framing MT as a route by which to improve learning or skill acquisition, MBAT may appeal to a wider array of trainees and professionals committed to improving their occupational performance.

The content and delivery of MBAT has been extensively tested and refined in a variety of high-stress professional environments including active-duty military personnel [34, 40, 43], and first responders [41]. Research results indicate that MBAT participation yields significant improvement in these populations on functional domains related to *attention* [40] *and related executive control functions, such as working memory* [44], *psychological health* [41], and *professional goals* [42]. Notably, the core content and practices remain the same during delivery to different high-stress populations, but program contextualization is adapted to the specifics of that population (for contextualization to medical student training, see Table 1). This feature helps to generate early awareness amongst participants of how the course is relevant to their professional and personal goals, thereby maximizing engagement with course content.

**Table 1:** General MBAT-Rx Structure. Each of MBAT's core themes is connected to a specific home practice and contextualized for medicine. Each theme is presented for 1 h. Participants are introduced to home practice during class sessions

**Class (2 hrs)**	**Core Theme**	**Home Practice**	**Contextualization**
1	**Focused Mind**: Optimizing performance personally and professionally **Brain Fitness**: Train your brain like your life depends on it	Mindful sitting (10 min)	Link between inattention and medical errors
2	**Attending**: Being present with your patient **Treat/Don’t Treat**: The Proportionate Response	Mindful body scan (15 min)	Training to be an “Attending” physician requires attention, focus, responses proportionate to situation
3	**Fear Factor**: Responding effectively in the face of fear **Embracing Uncertainly**: Thriving amidst uncertainty	Open monitoring (15 min)	Tying together how uncertainty in medicine can activate fear responses that degrade attention, paying attention mindfully can be an antidote
4	**The Power Within**: Expanding Interpersonal Capabilities **Ending and Beginning**: It only works if you work it	Connection practice (15 min)	Focus on interpersonal dynamics in medical teams, need for relational support in career and personal life

To facilitate ease of course delivery, each 2-hour class follows the same structure. Two specific themes are highlighted during each class session, with one hour dedicated to each theme (see Table 1 for specific themes in each class). To structure the hour, dedicated MBAT-Rx training cards are distributed at the start of each hour. The trainer begins by leading a short 3–5 mindfulness practice to help students settle in and refocus. The trainer then briefly introduces the theme of the hour using representative quotes presented on the training card. These quotes help facilitate a short 5–10-min discussion where students identify ways they have noticed that theme show up for them in medical training. After this discussion, students are directed to a short series of 3–5 questions which prompt them to consider their own reactions and reflections related to that theme. After a period of 5–10 min to quietly consider and write out responses to those questions, a trainer-led group discussion follows. This discussion is meant to be an exploration of students’ own insights and direct experience and serves as a way to introduce the upcoming mindfulness practice. The trainer then leads the students in a 15–20-min mindfulness practice which is targeted toward the theme of the prior discussions. The hour-long session concludes with a final 5–10-min debriefing session tied to their direct experience with the mindfulness practice they just completed. The second hour follows the same structure, using a new training card dedicated to the explicit theme for that hour.

Over the course of the two hours, participants will have engaged in approximately 40–50 min of explicit mindfulness practice. This training structure reinforces the importance of mindfulness practice, boosts students’ direct familiarity with practice, and provides an opportunity for the trainer to respond to any practice-related questions to ensure that students feel comfortable engaging in practice before doing it at home.

At the end of the two-hour session, participants are given a final training card to take home that summarizes the themes of the day and lists out the home practices to complete before the next class. Home practices for each week include the mindfulness practice that was first introduced and experienced during that class. Students are able to keep all training cards as reference material, and are encouraged to look back over them as needed to solidify insights.

### Training physicians to facilitate MBAT-Rx via the Train-the-Trainer (TTT) practicum

Another critical component of MBAT’s framing is the employment of context-familiar trainers who are able to relate the content of the MBAT program to the demands of individuals’ professions. Given recent evidence that delivery of MT via context-familiar vs unfamiliar trainers is a best practice for effective delivery of MT [34], medical school faculty may be best-positioned to deliver MT to medical students. In prior studies, context-familiar trainers received a 10- to 12-week MBAT trainer practicum in which they gained foundational knowledge of mindfulness concepts and practices and developed proficiency in delivering contextualized programs to their specific group. Individuals who have received MBAT delivered by a trainer who participated in this TTT model have been receptive to their mindfulness trainer [40], and have reported benefitting from the training [42].

MBAT’s development within professional training environments, focus on one's capacity to pay attention as foundational for professional development and psychological health, as well as the train-the-trainer dissemination model made it uniquely suited for the demands of the medical education environment. For this reason, in the current project, MBAT was contextualized for the medical school environment, and referred to as MBAT-RX.

Four medical school faculty members who are community-based physicians across four separate medical specialties, completed the TTT program. This program was organized around issues commonly faced in medical training. Having four trainers provided flexibility of delivery year-round, further increasing the feasibility for student participation. Physician faculty trainers were carefully selected to ensure that they were not concurrently involved in academic or clinical evaluation of first-year medical students. This consideration helped facilitate an environment of openness and ease among students. They could freely discuss difficulties without fear it may influence evaluation of their performance in other environments.

### Delivery of MBAT-Rx

Initial delivery of MBAT-Rx to medical students by trainers was offered in the Spring of 2018 to first-year medical students. This specific interval was chosen to provide students sufficient time to acclimate to the demands of medical school. Yet, it was also early enough in medical training to advantage their use of MBAT-related skills throughout their future education. Each year, students were first introduced to the course through an optional 20-min lunch time information session, where course instructors detailed the purpose and structure of the MBAT program. This session was advertised to the entire first year medical school class of around 140 students per year. Approximately 70 students from each class voluntarily signed up for the information session each year, indicating a high interest in this program across the medical school classes.

A total of 105 students enrolled in MBAT-Rx courses between 2018 and 2023. In 2018, the course was delivered twice, once early in the Spring Semester (February), and one later in the Spring Semester (April). Each of these courses had 15 students enroll, with approximately 7 students completing the program feedback in each course. Student feedback indicated that participants preferred the MBAT-Rx course offered earlier in the semester, as they were able to use the skills throughout the semester. To be responsive to this feedback, subsequent MBAT-Rx delivery was conducted in January or February, and it was only offered once per year. Between 2019–2023, 20–30 students enrolled in MBAT-Rx and 10–15 students completed the optional program evaluation responses each year, which resulted in 67 total students who provided feedback between 2018–2023. Since this was a program evaluation project and not a research study, no demographic information was collected.

While 2018 and 2019 cohorts experienced in-person training, a virtual format was required in 2020 and 2021 due to the COVID-19 pandemic. In-person delivery was resumed in 2022 and 2023. Remote and in-person delivery platforms resulted in similar enrollment numbers and program evaluation completion rates (10–15 students/year). Anecdotally, trainers felt that course discussions reached a deeper level during in-person years and underscored the importance of in-person training. In line with this feedback, once pandemic restrictions were lifted, the in-person format was resumed.

## Results

Medical education is a dynamic environment that continuously exposes students to novel programs aimed at improving their educational experience. Often, these programs are one-off experiences, and it can be difficult to understand the extent to which students feel the program benefitted their overall learning experiences. Program evaluations can therefore be useful to ascertain how valuable participants found the program by allowing them to provide feedback. This feedback can then be used to generate insights regarding aspects of the course that participants found more versus less useful, to guide program modifications before subsequent program delivery [45, 46].

The program evaluation of MBAT-Rx was conducted to glean program specific feedback versus advancing generalizable knowledge. While program evaluations are indeed informative, unlike formal research, they do not empirically investigate a research question, and therefore, research conclusions may not be made from findings derived from these evaluations [46]. Relatedly, the present program evaluation does not test a specific research question. The summary findings provided herein are intended to inform and improve future programs of short-form MT offered at medical schools. Before drawing empirical conclusions regarding MBAT in the medical school context, formal research of this program will need to be conducted.

With both remote and in-person delivery, MBAT-Rx is typically offered to class sizes of 20–30 students but can also scale to larger class sizes. The structure of the course, core themes, contextualization of the themes within medicine, and home practice assignments are detailed in Table 1.

**Fig. 1:**
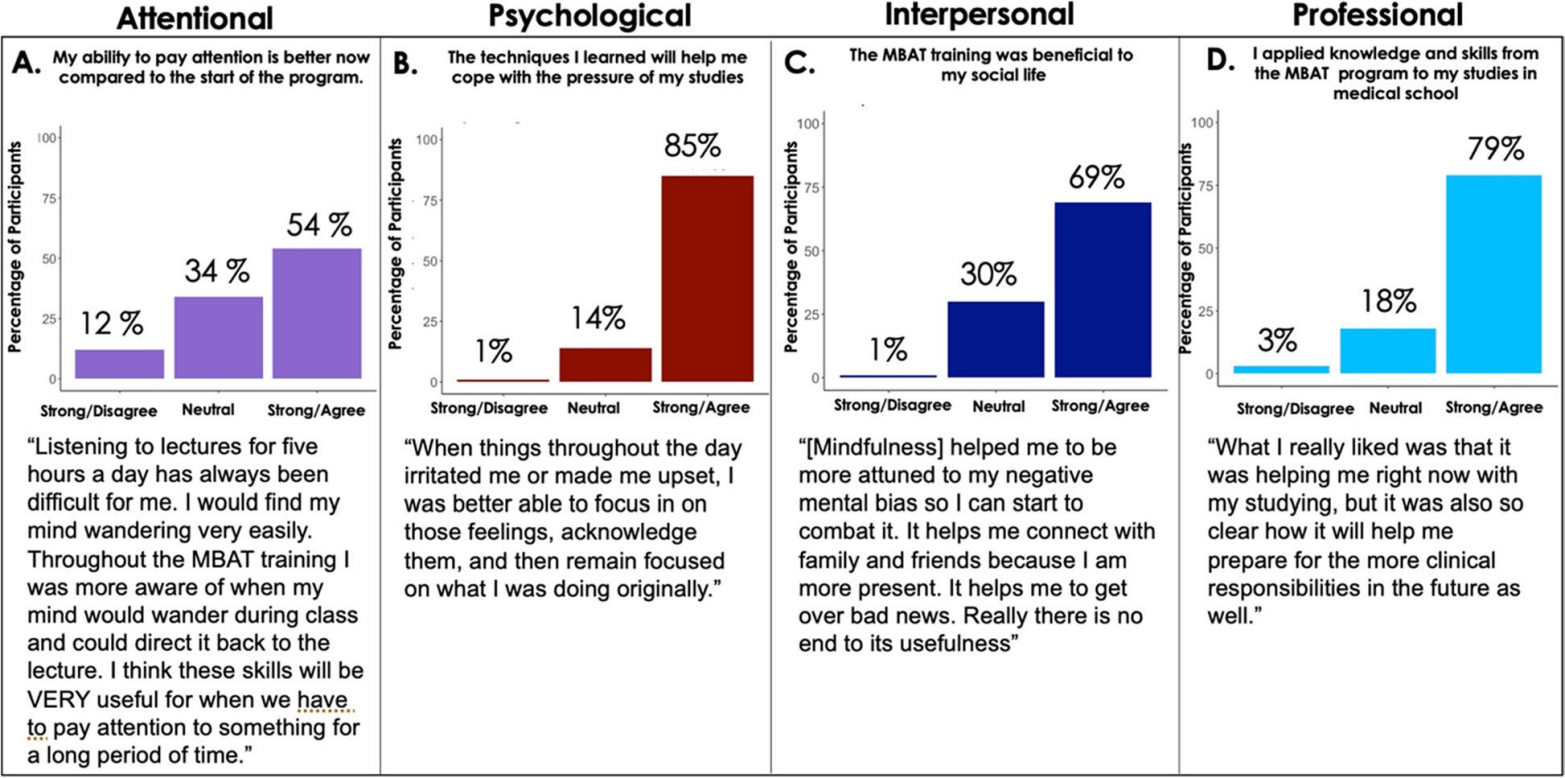
Student Experiences in MBAT-Rx across Functional Domains. Program evaluation of *n* = 67 students completing MBAT-Rx are presented in relation to (**A**) Impact on attention, (**B**) Impact on psychological health, (**C**) Impact on Interpersonal relationships, and (**D**) Impact on professional development. Representative quotes by students that speak to each domain are included to illustrate the subjective experience in the course. All responses to program evaluation questions were collected after the final MBAT-Rx class meeting

Students’ voluntary program evaluation responses indicated that they felt that MBAT-Rx addressed aspects of their life relevant to attention, psychological health, interpersonal function, and professionalism (see Fig. 1 for graphs and quotes).

**Fig. 2:**
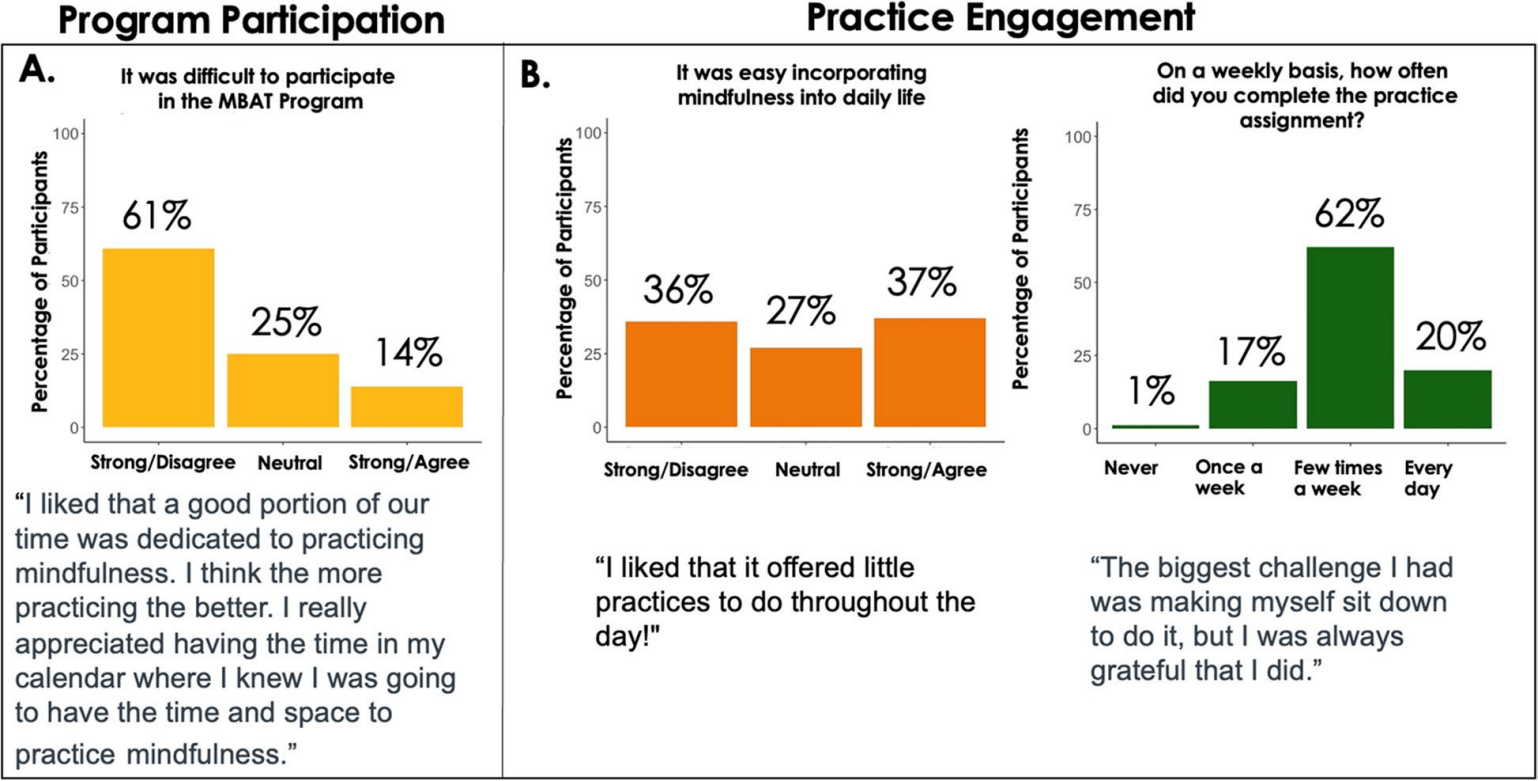
Student Perception of MBAT-Rx’s Time Demands. **A** The majority of *n* = 67 students in MBAT-Rx did not feel that the MBAT-Rx program was difficult to participant in. **B** Students reported a range of experiences in how easy it was to incorporate daily mindfulness practice, but the majority of participants (82%) reported practice at least a few times a week, despite the fact that 36% report finding mindfulness practice difficult to incorporate into daily life. All responses to program evaluation questions were collected after the final MBAT-Rx course

Program evaluation responses also indicated that MBAT-Rx successfully addresses the two main structural challenges of time demands and contextualization. In terms of time demands, students reported they were able to meet the time requirements for the weekly class meeting, as well as incorporate the daily practice requirements into their daily schedules, with the majority (82%) indicating that they completed the home practices from a few times a week to every day (Fig. 2b). It is important to note that while the majority of students (61%) reported that it was not difficult to participate in the overall MBAT-Rx program (Fig. 2a), 36% of students reported they did not find it easy to incorporate mindfulness into daily life (Fig. 2b). This indicates that students were able to complete the allotted home practice time (10-15 minutes), but did not necessarily find it easy to do so. Because the broader MT literature reports that greater practice engagement corresponds with greater benefits for participants [40], trainers consistently emphasized the importance of finding the time to do home practice even when it feels difficult. They checked in with students each week about it to provide motivation and support. This attention to practice engagement may be one reason why the majority of students reported completing the home practice at least a few times a week, despite finding it difficult to do so (Fig. 2b).

**Fig. 3:**
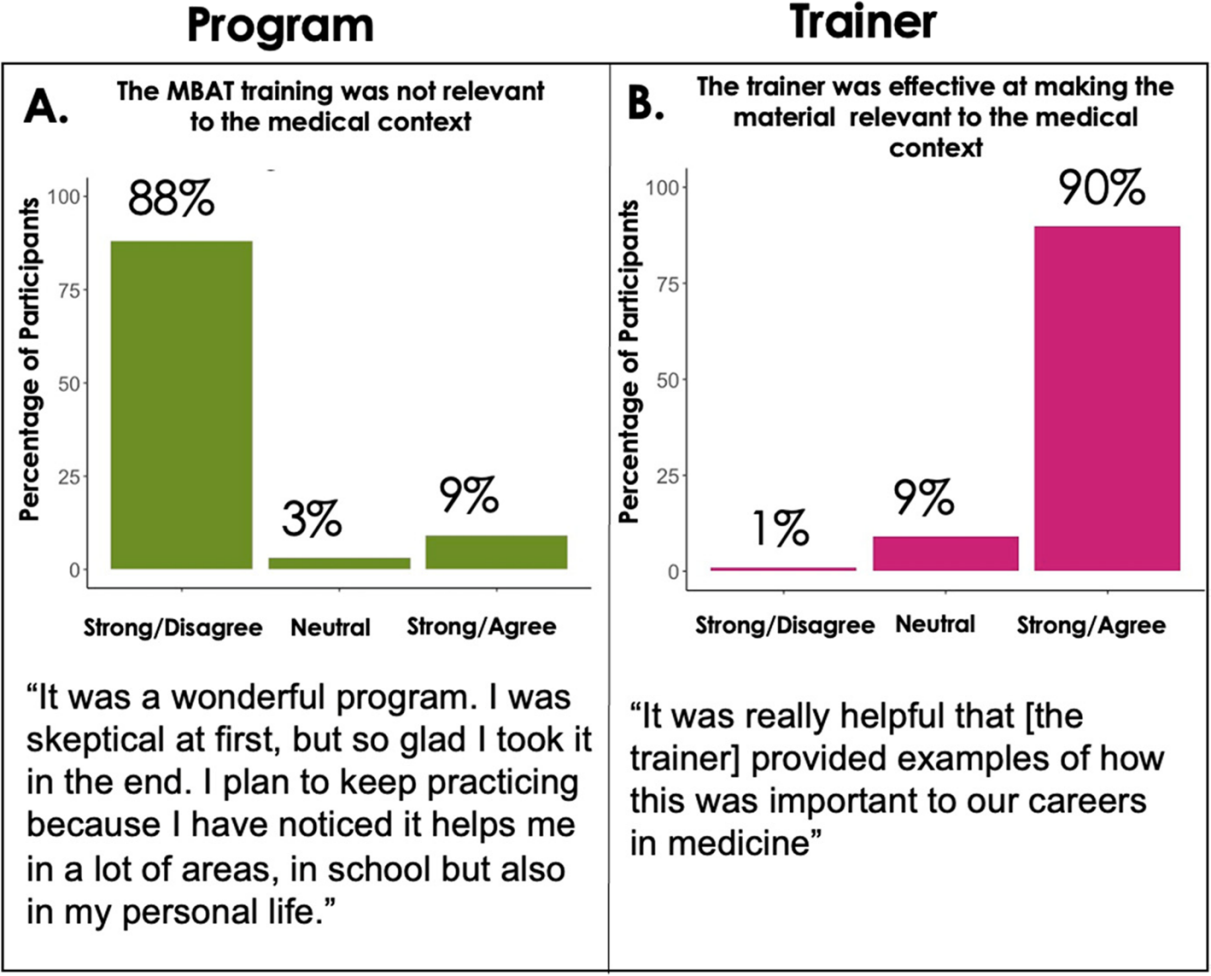
Student Perception of MBAT-Rx’s Contextualization to Medicine. **A** The majority of *n* = 67 students completing MBAT-Rx felt the program was relevant to the medical school context. **B** Students strongly felt that the physician faculty trainers were effective at relating the material to the medical context. All responses to program evaluation questions were collected after the final MBAT-Rx class meeting

In terms of contextualization, students reported that the program was relevant to their goals in medicine and felt the knowledge and skills they gained would improve their performance in medicine (Fig. 1d, and 3).

The train-the-trainer program was crucial for contextualizing MBAT-Rx to the medical school context and simultaneously meeting trainees’ goals across functional domains (Fig. 3). Students responded extraordinarily well to the physician faculty trainers. Students’ typical interactions with faculty in their first year of medical school primarily entails evaluation, leaving little room for informal discussion and mentorship. Given this, some students expressed appreciation for the opportunity to engage with their faculty trainers and openly discussing their difficulties and triumphs of medical training. The small group format and conversations guided by the trainers and the MBAT-Rx training cards further fostered a sense of camaraderie and facilitated a supportive environment amongst peers, which can be difficult to achieve in wellness programs for medical students.

## Discussion

While our initial pilot implementation of MBAT-Rx only examined voluntary program evaluation responses, participants broadly indicated that MBAT-Rx may not only surmount the barriers commonly faced by MT programs in medical training environments, but they also found the program helpful for improving their attention, psychological, interpersonal, and professional functions. Their responses indicated that MBAT-Rx’s framing may successfully utilize mindfulness practices to link academic, professional, and personal flourishing. Future systematic research involving objectively indexed MT-related neurocognitive performance changes, as well as psychological health changes indexed via self-report, relative to controls, is necessary (see Jha et al., 2007) [31]. Additionally, systematic investigations of the impact of MT on objective measures of medical school performance (e.g., exam grades or standardized patient physical exams, OSCEs) may be important for linking participation in MBAT-Rx to metrics of academic and professional development that students value. Such work could underscore if and how the practice of mindfulness is both protective of wellness and cognitive health throughout high-stress pre-professional training, while also bolstering professional development.

It is important that MT programs are understood as one approach among many to counter medical students' distress and facilitate professional development. Mindfulness interventions alone cannot eradicate the institutional challenges endemic to medical education that pose detrimental consequences on medical students’ personal and professional lives. Structural changes that foster an optimal learning environment are necessary to promote medical student flourishing (e.g., pass/fail grading, implementation of duty hour restrictions, regulation of 24-h call shifts, etc.) [39, 47]. MT may represent one step towards promoting such changes, as it allows students to become aware of their circumstances with steadiness versus reactivity, and attend to thoughts and emotions in a way that can elicit the most effective actions and long-term change.

While the program feedback presented here suggests the utility of short-form mindfulness programs such as MBAT-Rx, there are a number of notable limitations. First and foremost, due to this simple program evaluation format, we did not collect comprehensive demographic information from participants. We, therefore, cannot gain meaningful insights into factors that may differ between those who decided to enroll versus those who did not, and secondarily into those who completed the program and program evaluations versus those who did not. Of a medical school class size around 140, approximately 70 students voluntarily attend the MBAT-RX info session, indicating a high degree of student interest in the course content. However, only 20–30 students typically enrolled in MBAT-Rx per year, which is a relatively low percentage of total students. Course scheduling may have influenced this. MBAT-Rx was offered one day a week, which could have conflicted with other events, and precluded more students from participating. One solution to this going forward is to offer multiple simultaneous sessions of MBAT-Rx on different days/times to maximize the number of students who can enroll.

Another major limitation is that we did not track engagement with home practices. Students were sent MP3 recordings of the weekly mindfulness practice after each class. Future research studies should track participants’ engagement with mindfulness practices. This would allow researchers to ascertain the amount of practice necessary to confer salutary effects on a particular outcome. Delivery of recordings via a smartphone application, with daily notification reminders, could be another way of both tracking practice engagement and also encouraging formation of a daily practice habit. The program evaluation indicated that some students struggled to incorporate mindfulness into their daily lives. Future research should investigate the extent to which this perceived difficulty influences students’ engagement with mindfulness practices.

One major drawback of the feedback format was that it did not include long-term follow-up questions to assess continued engagement with mindfulness practices after course completion. Importantly, at the end of the course, students were encouraged to continue their practice by seeking out weekly drop-in mindfulness offerings hosted by students at the medical school, or setting up a “buddy-system” with classmates to hold each other accountable. Tracking the extent to which students utilize these suggestions and the effect of such continued engagement on measures of interest, will be important for establishing the impact of MT on medical students over time.

The generally positive feedback of MBAT-RX presented here is meant to motivate future research to understand the impact of short-form MT on the cognitive, emotional, and professional development of medical students. Research measures of interest could assess effects on academic performance such as exam scores, or on clinical assessments of patient care via OSCE assessments. Established measures of attentional, psychological, or interpersonal health, as well as stress, resilience, and burnout may be additionally important. Neurocognitive effects could be assessed with EEG or MRI during laboratory-based tasks. It will be very important to systematically assess such effects against classmate controls who do not engage in MBAT-Rx and to include plans for long-term follow-up.

Overall, our implementation of MBAT-Rx to medical students in their first year suggests this short-form MT program can integrate seamlessly into the existing structure of medical education and is well-adapted to the medical context. While student feedback indicated that MBAT-Rx did address wellness domains, it is notable that it also appeared to improve core competencies relevant to the educational goals of becoming an attending physician. We focused specifically on delivery of MBAT-Rx to first-year medical students to foster protective/preventive skill training at the outset of medical training. MBAT-Rx can be easily adapted to later delivery in medical school or residency that focuses more on trainees’ experiences during the intense clinical years. Future work can explore the offering of additional “booster” sessions to promote mindfulness practice along the continuum of medical education as well.

## Conclusion

As medical schools continuously refine medical curricula to promote the highest quality of medical education, we argue that mindfulness offerings have a valuable role to play not only in addressing and preventing distress during training, but also, in nourishing a professional identity that takes into account the whole person. By contextualizing mindfulness offerings to the specific goals of each stage of medical education, MBAT-Rx appeals to a wide range of medical students. In offering mindfulness courses at convenient times within training, schools can more effectively engage a wide array of students. Future efforts to incorporate MT into medical curricula or wellness initiatives may thus benefit from using MBAT-Rx or similar programs that emphasize and employ the principles of contextualization, and time efficient delivery by physician faculty trainers.

## Data Availability

No datasets were generated or analysed during the current study.

## Abbreviations

MBAT: Mindfulness Based Attention Training
MBAT-Rx: Mindfulness Based Attention Training - medical student context
MT: Mindfulness Training
TTT: Train-the-Trainer dissemination model
